# Protein Kinase D Is Dispensable for Development and Survival of *Drosophila melanogaster*

**DOI:** 10.1534/g3.119.400307

**Published:** 2019-05-29

**Authors:** Dieter Maier, Anja C. Nagel, Alexandra Kelp, Anette Preiss

**Affiliations:** Universität Hohenheim, Institut für Genetik (240A), Garbenstr. 30, 70599 Stuttgart, Germany

**Keywords:** *Drosophila melanogaster*, Protein kinase D, *PKD* null mutant, oxidative stress response, redundancy

## Abstract

Members of the Protein Kinase D (PKD) family are involved in numerous cellular processes in mammals, including cell survival after oxidative stress, polarized transport of Golgi vesicles, as well as cell migration and invasion. PKD proteins belong to the PKC/CAMK class of serine/threonine kinases, and transmit diacylglycerol-regulated signals. Whereas three PKD isoforms are known in mammals, *Drosophila melanogaster* contains a single PKD homolog. Previous analyses using overexpression and RNAi studies indicated likewise multi-facetted roles for *Drosophila* PKD, including the regulation of secretory transport and actin-cytoskeletal dynamics. Recently, involvement in growth regulation has been proposed based on the hypomorphic d*PKD^H^* allele. We have generated *PKD* null alleles that are homozygous viable without apparent phenotype. They largely match control flies regarding fertility, developmental timing and weight. Males, but not females, are slightly shorter lived and starvation sensitive. Furthermore, migration of pole cells in embryos and border cells in oocytes appears normal. *PKD* mutants tolerate heat, cold and osmotic stress like the control but are sensitive to oxidative stress, conforming to the described role for mammalian PKDs. A candidate screen to identify functionally redundant kinases uncovered genetic interactions of *PKD* with *Pkcδ*, *sqa* and *Drak* mutants, further supporting the role of PKD in oxidative stress response, and suggesting its involvement in starvation induced autophagy and regulation of cytoskeletal dynamics. Overall, PKD appears dispensable for fly development and survival presumably due to redundancy, but influences environmental responses.

## Introduction

**Figure 1:**
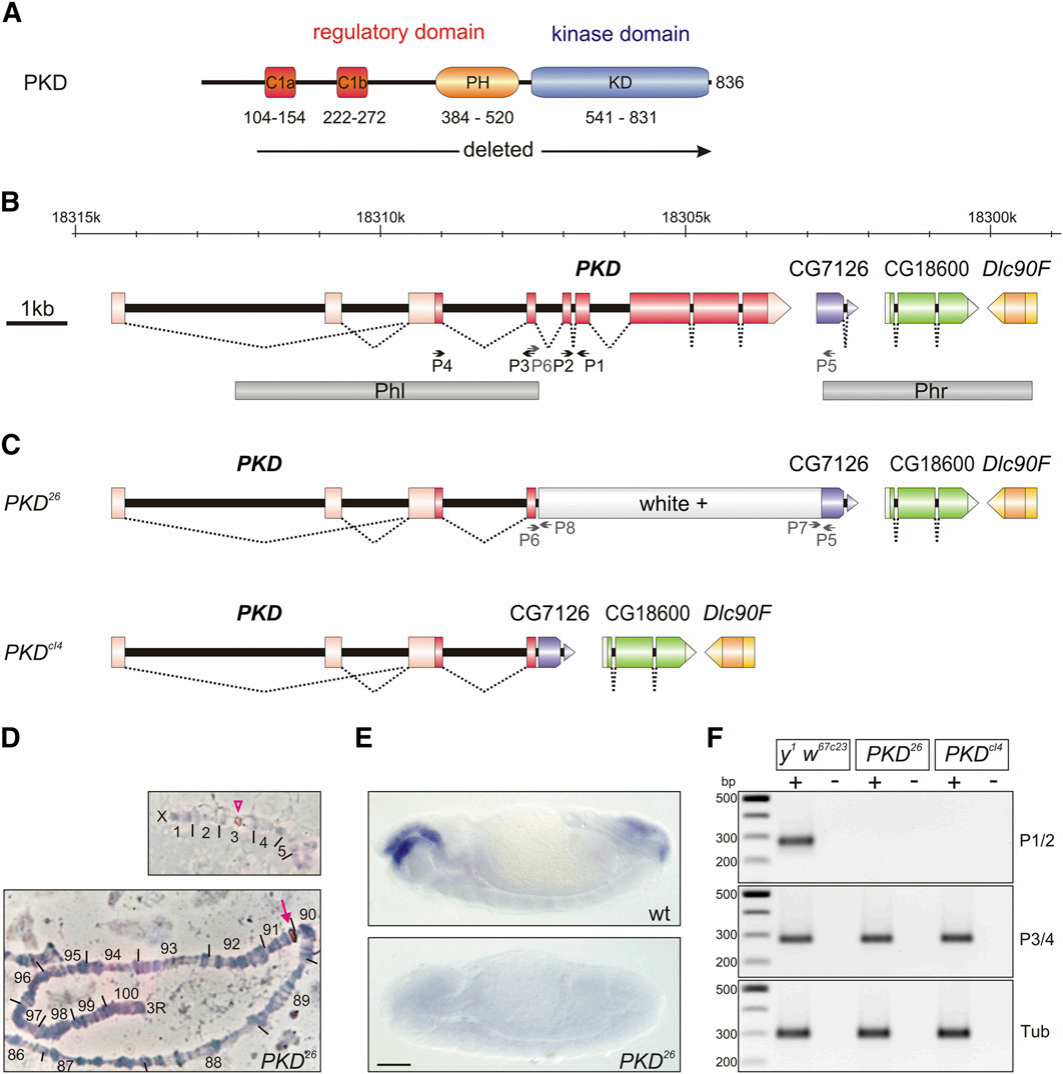
Generation of *PKD* mutants by homologous recombination. (A) Scheme of the PKD protein; it comprises 836 amino acids. The protein is subdivided in a regulatory and a catalytic kinase domain (KD). Characteristics of the regulatory domain are tandem zinc-finger motifs (C1a, C1b), and a pleckstrin homology domain (PH). Numbering depicts codons. The deletion generated in the mutants starts within codon 91, and hence affects all relevant domains of the PKD protein. (B) Genomic map of the *PKD* locus and the proximal genes CG7126, CG18600 and *Dlc90F* (numbers give sequence location according to flybase). Exons are shown as boxes, coding exons are shaded darker. Underneath, the two DNA fragments (Phl, Phr) used for homologous recombination are shown. P1-P6 depicts roughly the position of primers used in confirmation experiments (not to scale). No function has yet been assigned to CG7126 or CG18600. (C) Map of *PKD^26^* and *PKD^cl4^* mutants. In the *PKD^26^* mutant, most of the *PKD* locus was replaced by the white^+^ marker gene, which was deleted in *PKD^cl4^* by Cre-loxP mediated recombination. P5-P8 depicts roughly primer positions (not to scale). (D) Confirmation of the successful homologous recombination event in the *PKD^26^* allele. *In situ* hybridization with a white*^+^* probe on salivary gland chromosomes from *PKD^26^* mutants highlights the *white* locus at the tip of the X-chromosome in the upper panel (open arrowhead), and shows insertion of the *w*^+^-bearing transgene at position 91A on the right arm of the third chromosome (closed arrow). Numbers correspond to chromosomal sections (Lindsley and Zimm 1992). The whole spread is shown in supplemental Figure S1. (E) *In situ* hybridization of a *PKD* probe on whole mount wild type (wt, upper panel) and homozygous *PKD^26^* embryos (lower panel) confirms absence of *PKD* transcripts in the mutant. Scale bar corresponds to 100 µm for both panels. (F) RT-PCR was performed on RNA of *PKD^26^* and *PKD^cl4^* mutant and *y^1^ w^67c23^* control flies as indicated. (+) with reverse transcriptase and (-) no-RT control. Primer pairs P1/P2 (278 nt) and P3/P4 (282 nt) (see (A)) overlap the fifth and the third introns, respectively. No transcripts beyond exon four are detected in the *PKD* mutant animals. Tubulin primers served as control for intact mRNA (Tub, expected 299 nt). *NEB 100 bp DNA-ladder* served as size standard (500 bp reference, 300 bp and 200 bp are labeled). The picture was inverted for better visibility.

Protein Kinase D (PKD) isoforms are serine/threonine kinases of the Protein Kinase C family typified by a long N-terminal regulatory region followed by a catalytic kinase domain (Figure 1A). The regulatory region contains two cysteine-rich domains that bind to diacylglycerol and phorbolesters, and a Pleckstrin-homology module, that auto-inhibits the catalytic domain (Figure 1A) (reviewed in Fu and Rubin 2011). Once recruited to the membrane by diacylglycerol, PKD is activated by members of the PKC family through the phosphorylation of two serine residues in the activation loop of the kinase domain (Fu and Rubin 2011). Based on sequence similarity of the kinase domain, PKD has been classified as member of the Ca^2+^/Calmodulin-dependent serine/threonine protein kinases (CAMK) (Fu and Rubin 2011, Cobbaut and Van Lint 2018).

Three PKD isoforms exist in mammals, where they are involved in various processes, including the regulation of a cell survival response upon oxidative stress, cell proliferation, cell differentiation, cell motility and invasion, as well as secretory transport from the *trans*-Golgi compartment to the plasma membrane (reviewed in Fu and Rubin 2011, Olayioye *et al.* 2013, Cobbaut and Van Lint 2018). The extent of overlap in their biological function is still a matter of debate, but it is evident that all three act as multi-functional kinases with a major role in structural integrity and function of the Golgi complex as well as in the regulation of actin-cytoskeletal dynamics (Fu and Rubin 2011, Olayioye *et al.* 2013). Interestingly, a knock-out mutation in the murine PKD1 gene allows normal mouse development and fertility, albeit affecting oxidative stress response in embryonic fibroblasts (Zhang *et al.* 2015). Whereas this result may be taken as indication for functional redundancy of the three PKD isoforms, it may also be interpreted as a highly (perhaps cell type) specific, non-lethal function of the respective kinase. Alternatively, only laboratory, non-stressed conditions may allow normal development (Zhang *et al.* 2015).

*Drosophila melanogaster* harbors a single *PKD* homolog with a similarity of 67% and identity of nearly 60% to any of the three human PKD kinases (Maier *et al.* 2006). Accordingly, *Drosophila* has been used as an *in vivo* model to study the biological roles of PKD. The *Drosophila PKD* gene is broadly expressed throughout development. Whereas PKD mRNA is uniform in imaginal tissues, it strongly accumulates in ectodermal derivatives in the late embryo, detected for example in the epithelia of the epidermis, the salivary glands, the hind- and the foregut (Maier *et al.* 2006). A ubiquitously expressed PKD-GFP fusion protein was present in the cytosol and along cell membranes, and in the *trans*-Golgi compartment of secretory tissues like the salivary glands (Maier *et al.* 2006). The biological function of PKD in the fly was assessed by the overexpression of presumptive activated and dominant negative isoforms of PKD, as well as by RNA interference experiments (Maier *et al.* 2006, Maier *et al.* 2007, Nagel *et al.* 2010). Whereas the overexpression of a dominant negative PKD isoform interfered with pattern formation in the wing, the activated PKD-SE isoform affected development more broadly (Maier *et al.* 2007). RNAi-mediated knock-down of PKD activity effected tissue loss primarily through apoptosis. Interestingly, a light-dependent degeneration of the adult retina was observed in flies overexpressing activated PKD-SE (Maier *et al.* 2007). Being a typical consequence of rhodopsin maturation or trafficking defects (Colley *et al.* 1995), this phenotype points to a role of PKD in secretory transport and in cytoskeletal dynamics (Maier *et al.* 2007). The latter aspect was corroborated by the finding that *Drosophila* PKD, like its mammalian counterpart PKD1, impacts actin remodelling by the regulation of cofilin activity through its phosphatase Slingshot (Barišić *et al.* 2011). Accordingly, accumulation of F-actin and phosphorylated cofilin was likewise observed in cell clones either overexpressing activated PKD-SE or lacking Slingshot (Nagel *et al.* 2010, Barišić *et al.* 2011). These investigations provided evidence for a possible role of *Drosophila* PKD in cell motility by modulating actin dynamics (Barišić *et al.* 2011). Recently, a role of *Drosophila* PKD in the secretion of insulin like peptide ILP2 was uncovered, thereby influencing metabolism and growth of the developing animal (Ashe *et al.* 2018). In sum, *Drosophila* PKD appears to be a multifunctional kinase like its mammalian homologs (Fu and Rubin 2011, Olayioye *et al.* 2013).

In order to address the role of *Drosophila* PKD in further detail, we generated null alleles by ends-out directed homologous recombination (*PKD^26^*, *PKD^cl4^*). Both alleles turned out to be homozygous viable without apparent phenotypes. With regard to developmental timing, life span, fertility, weight and fat content, the *PKD* null mutants were largely within the range of the control. Whereas the *PKD* null mutants tolerated various stress factors, sensitivity toward oxidative stress was uncovered. In a candidate kinase screen, we obtained evidence for redundant kinase function regarding oxidative stress response, starvation induced autophagy and regulation of cytoskeletal dynamics. Overall, our data indicate that PKD is largely dispensable for development and survival of *Drosophila* but is required for combatting oxidative stress.

## Materials and Methods

### Generation and confirmation of PKD mutant alleles

To generate *PKD* null mutants, we followed the ‘ends-out’ homologous recombination protocol developed by Gong and Golic (2003). To this end genomic fragments covering 5′ and 3′ regions of the locus were PCR-isolated from lambda phage ED clones (Maier *et al.* 1992): a 5 kb genomic *Acc*65I/*Asc*I fragment starting within the first intron and covering the second coding exon (Phl; Figure 1B), and a 3.4 kb *Acc*65I/*Sph*I fragment overlapping the downstream CG18600 locus (Phr; Figure 1B). Primers included restriction enzyme target sites for cloning (bold):

PKDPhlUP 5′ **GGT ACC** GCA ATA TGC CGC TGT TAT TTA TTG ATC AAT 3′

PKDPhlLP 5′ **GGC GCG CC**T TAC GAC TGG TGG TCA GCA CGA TTT C 3′

PKDPhrUP 5′ **GGT ACC** GCG GGA GAG ATT CTG TAT GAG CAG TA 3′

PKDPhrLP 5′ **GCA TGC** CCA AAA ACG CGC GCA CAT TTA CAA C 3′

Fragments were cloned into pW25 transformation vector using compatible restriction sites (Gong and Golic 2003) to generate transgenic starter line T15-2 (second chromosomal insertion) by classical *P*-element mediated germ line transformation of *y^1^ w^67c23^* flies (Rubin and Spradling 1982). Homologous recombination was performed as described before (Gong and Golic 2003, Fischer *et al.* 2015), starting with 592 G1 single-crosses resulting in the line *PKD^26^*. The inserted *white* marker gene was excised with help of I-*Cre*I exactly as described before (Rong *et al.* 2002) to yield allele *PKD^cl4^*.

Homologous recombination at the *PKD* locus at 92E was confirmed for *PKD^26^* by chromosome *in situ* hybridization, by Southern blotting as described earlier (Preiss *et al.* 1988), and by PCR with primer pairs P1/2, P3/4, P5/6, P5/7 and P6/8. Loss of mRNA expression in *PKD^26^* was confirmed by *in situ* hybridization on whole mount embryos according to standard protocols (Tautz and Pfeifle 1989) and by RT-PCR. The breakpoint fragment generated by PCR from *PKD^cl4^* total DNA with primer pair P5/P6 was sequence confirmed. The following primers were used (position is schematically shown in Figure 1B):

P1 M-UP 5′ TCG AGT CCT CCG TGG AGA CGA 3′

P2 M-LP 5′ CTC CGA GAT GCC GAC CCT CAA 3′

P3 5′RT-UP 5′ GGC GGT CAG CAC GAT TTC CA 3′

P4 5′RT-LP 5′ AGC GTT CCC GTT ATC ATG GAG 3′

overlaps *PKD^cl4^* deletion

P5 Cre-lox UP 5′ ACC CCA ACT TCC TCA TCT TC 3′

P6 Cre-lox LP 5′ CCG GAC AGT GGA CTC ACA TA 3′

overlaps *PKD^cl4^* deletion, used for cloning/sequencing of the breakpoint fragment

P5 PKD Cre-lox UP clone 5′ GGT TGA GCT GGA TGA ATG TTT C 3′

P6 PKD Cre-lox LP clone 5′ GGA ACG CAT TCT CCT CTT CGT C 3′

with P5 Cre-lox LP

P8 white UP 5′ AAA AGT GCA GCG GAA ATA GTT A 3′

with P6 Cre-lox UP

P7 white LP 5′ ACG CTA TCG ACC TAT TCA GA 3′

Tubulin56D primers

Tub56D-229UP 5′ GAA CCT ACC ACG GTG ACA GCG A 3′

Tub56D-507LP 5′ GAA GCC AAG CAG GCA GTC GCA 3′

### In situ hybridization

Polytene chromosomes from salivary glands of *PKD^26^* homozygous mutant third instar larvae were prepared for *in situ* hybridization as outlined in Ashburner (1989). The probe, labeled with DIG-dUTP by random priming (Tautz and Pfeifle 1989), was generated using CaSpeR-vector as template as it contains the white^+^ minigene (Pirrotta 1988), using the DIG DNA Labeling and Detection kit (Roche; Merck). Hybridization was as outlined in Ashburner (1989), and detection according to the manufacturer’s protocol. *In situ* hybridization on whole mount embryos was as described earlier (Tautz and Pfeifle 1989; Maier *et al.* 2006). Embryos from *y^1^ w^67c23^* control flies and *PKD^26^* mutant flies were collected overnight. As probe, we used *PKD* cDNA (pOT GH26429) (Maier *et al.* 2006), labeled with DIG-dUTP as above.

### Transcriptional analysis by RT-PCR

Poly(A)^+^ RNA was isolated with the *PolyATract System 1000 kit* (Promega Mannheim, Germany) from 25 *PKD^26^*, *PKD^cl4^* and *y^1^ w^67c23^* male flies each according the supplier’s protocol. cDNA was produced with *qScriber cDNA Synthesis Kit* (highQu, Kraichtal, Germany) according to the manufacturer’s protocol. Amplification was with primer pairs P1/P2 and P3/P4, both overlapping introns: Genomic DNA should yield a 352 bp and a 1662 bp amplificate, respectively, whereas 278 bp and 282 bp are expected from cDNA. Tubulin 56D primers served as positive control (see primer list above). Absence of genomic DNA was tested in a non-RT control. NEB quick-load 100 bp DNA ladder was used as size standard.

Real time qRT-PCR was conducted as outlined in Praxenthaler *et al.* (2017) using *Blue S’Green qPCR kit* (Biozym, Hessisch-Oldendorf, Germany) on 10 ng of cDNA from *PKD^26^*, *PKD^cl4^* and OreR flies in 10μl end volume using MIC magnetic induction cycler (*bms*, Pots Point, Australia) including target, no-template and non-RT controls. As internal references for *PKD* or SOD expression, *βTub56D* and *tbp* were used. Primers were selected from the DRSC FlyPrimer bank (Hu *et al.* 2013): *SOD1*, PP70435; *SOD2*, PP70435; *tbp*, PP1556. Primers for *Tub56D* and *PKD* (P1/P2) are listed above. Relative quantification of three biological and two technical replicates was performed with *micPCR* software Version 2.6.5 based on *REST* taking target efficiency into account (Pfaffl *et al.* 2002).

### Fly stocks

Information on strains is available at https://flybase.org. Crosses, combinations and recombinations were performed with standard genetic techniques. Double mutants were confirmed by PCR. The following stocks were used: Oregon R (OreR) and *y^1^ w^67c23^* (BL6599), *w^1118^* either isogenic line BL5905 or BL6326, Canton-S (CS), *p38a^1^* (BL8822), *bsk^1^* (BL3088), *aPKC^k06403^* (BL10622), *Drak^BG00876^* (Bellen *et al.* 2004), *par-1^k06323^* (BL10615), *Pkcdelta^e04408^* (BL18258), *sqa^f01512^* (BL18446), *Strn-Mlck^c02860^* (BL11089), Df(2R)Exel6065, Df(1)Exel6227, Df(1)Exel6236, Df(2L)Exel7077 (Parks *et al.* 2004).

### Phenotypic analyses

Flies were raised under non-crowded conditions on standard agar-corn-molasses food (per liter 18g dry yeast, 10g soy flour, 22g molasses, 80g malt extract, 80g cornmeal, 6.25ml propionic acid, 8g agar-agar) at constant 25° and 78% humidity. Analyses were performed on one to five days old flies. To investigate *developmental timing*, offspring from parallel *inter se* crosses of 5 females and 3 males each was counted at days 8 to 18 (Wang *et al.* 2003, Fischer *et al.* 2015). To determine *fertility*, 4-8 virgin females (one to four days old) were kept in a vial with wild type males for three days, and then put on fresh food for 4-5 days at 18°; the number of offspring was recorded and calculated per female per day on food. For *longevity* experiments, animals separated by sex at the day of eclosion were transferred in batches of 25-30 to fresh food every third day; dead animals were recorded daily (Clancy *et al.* 2001, Wang *et al.* 2003, Tettweiler *et al.* 2005). High sucrose medium contained additional 10% sucrose (Wang and Clark 1995; Magwere *et al.* 2004). Using a precision balance *adult weight* was determined in batches of five animals 1-2 days after hatching (Fischer *et al.* 2015). Relative *fat content* was determined as percentage of dry weight as outlined in Vermeulen *et al.* (2006). The *larval floating* test was applied on fully fed, third instar wandering larvae in 8%, 10% and 12% sucrose in PBS, respectively (Reis *et al.* 2010; Fischer *et al.* 2015). *Resistance to starvation* was recorded either in the absence of food (wet starvation) or absence of all (dry starvation). To this end, sexed flies (2-3 days old) were starved in batches of 25 in empty vials (dry starvation) or in vials supplemented daily with a wet filter paper (wet starvation); dead animals were recorded regularly (Craig *et al.* 2004; Wang *et al.* 2004). *Thermotolerance* of flies, collected 2 days after eclosion, was tested in dry heat, wet heat and cold. Survival was determined by incubating flies in batches of 25-30 at 37° in an incubator on pre-warmed food for 2-3 hr (dry heat), or alternatively by submersing vials in a 37° degree water bath and recording dead animals regularly (Craig *et al.* 2004, Nielsen *et al.* 2005). To test cold sensitivity, two days old animals were cooled to 4° for 2 hr and recovery time until walking was recorded (Nielsen *et al.* 2005). To generate *oxidative stress*, 20 males 3-5 days old were first starved for four hours on 1% agarose/PBS medium, and then transferred to vials with filter paper soaked with a solution of 20 mM paraquat in 5% sucrose at 25° in the dark; dead flies were recorded twice a day, and live flies then transferred to a fresh paraquat containing vial (Clancy *et al.* 2001; Wang *et al.* 2003). *Osmotic stress* was applied by rearing flies on food containing additional 0.5 M NaCl; death toll was recorded daily (Craig *et al.* 2004).

Border cell migration was studied by staining ovaries with rhodamine-coupled phalloidin (Molecular Probes, Eugene OR, USA) as outlined before (Nagel *et al.* 2012), analyzed by confocal microscopy using a BioRad MRC1024 coupled to a Zeiss Axiophot and LaserSharp 2000 imaging software (Carl Zeiss AG, Oberkochen, Germany). Pole cells were stained in embryos using anti-vasa antibodies (developed by A. C. Spradling and D. Williams, obtained from the Developmental Studies Hybridoma Bank (DSHB) developed under the auspices of the NICHD and maintained by the Univ. of Iowa, Dept of Biology, Iowa, USA) as outlined before (Hay *et al.* 1988). Goat secondary antibody coupled to alkaline phosphatase was obtained from Jackson Immuno-Research Laboratories (Dianova, Hamburg, Germany). Microphotographs of chromosomes, embryos, larvae or adults were taken with a Pixera ES120 digital camera (Optronics) coupled to a Zeiss Axiophot or to a Leica Wild M3C stereomicroscope using the Pixera Viewfinder Version 2.0 software. Figures were assembled using *Corel Photo Paint*, *Corel Draw*, *Exel*, and *BoxPlotR* software. Statistical significance of probes was determined by ANOVA two-tailed test for multiple comparisons using Dunnet’s approach with raw p-values: *P* > 0.05 (not significant); * *P* < 0.05; ** *P* < 0.01; *** *P* < 0.001.

### Data availability

*PKD* mutant strains are available upon request. Supplemental data comprise 4 Supplemental Figures S1 to S4 in one file and one Supplemental Table S1. Figure S1 shows the complete chromosome spread of Figure 1D. Figure S2 contains the comparison of the developmental timing of *PKD* mutants and several additional controls. Figure S3 contains the comparison of the lifespan of *PKD* mutants and several additional controls. Figure S4 contains the comparison of the starvation resistance of *PKD* mutants and several additional controls. Table S1 contains details on the kinase mutants used in the candidate screen. Supplemental material available at FigShare: https://doi.org/10.25387/g3.7667078.

## Results

### Generation of PKD null mutant alleles by ends-out homologous recombination

The *Drosophila PKD* locus has been mapped to the right arm of chromosome 3 at position 91A2 (https://flybase.org). In order to generate specific *PKD* mutant alleles, we employed the technique of ends-out homologous recombination (Figure 1B, C) (Gong and Golic 2003). In allele *PKD^26^*, all relevant coding regions of *PKD* were replaced by the *white^+^* gene used for selection, *i.e.*, the regulatory and the catalytic domain of the kinase (Figure 1A-C). Replacement of the *PKD* locus by the *white^+^* gene in *PKD^26^* was confirmed by chromosomal *in situ* hybridization (Figure 1D, supplemental Figure S1). In allele *PKD^cl4^*, the *white* gene was deleted by Cre I-mediated recombination as outlined before (Rong *et al.* 2002). The resultant deletion was confirmed by sequence analysis of a PCR amplificate overlapping the breakpoint (Figure 1B, C). Absence of PKD transcripts was verified by *in situ* hybridization on whole mount *PKD^26^* embryos (Figure 1E) and by RT-PCR (Figure 1F) and qRT-PCR for both alleles, respectively.

### PKD null mutants are homozygous viable without apparent phenotype

**Figure 2:**
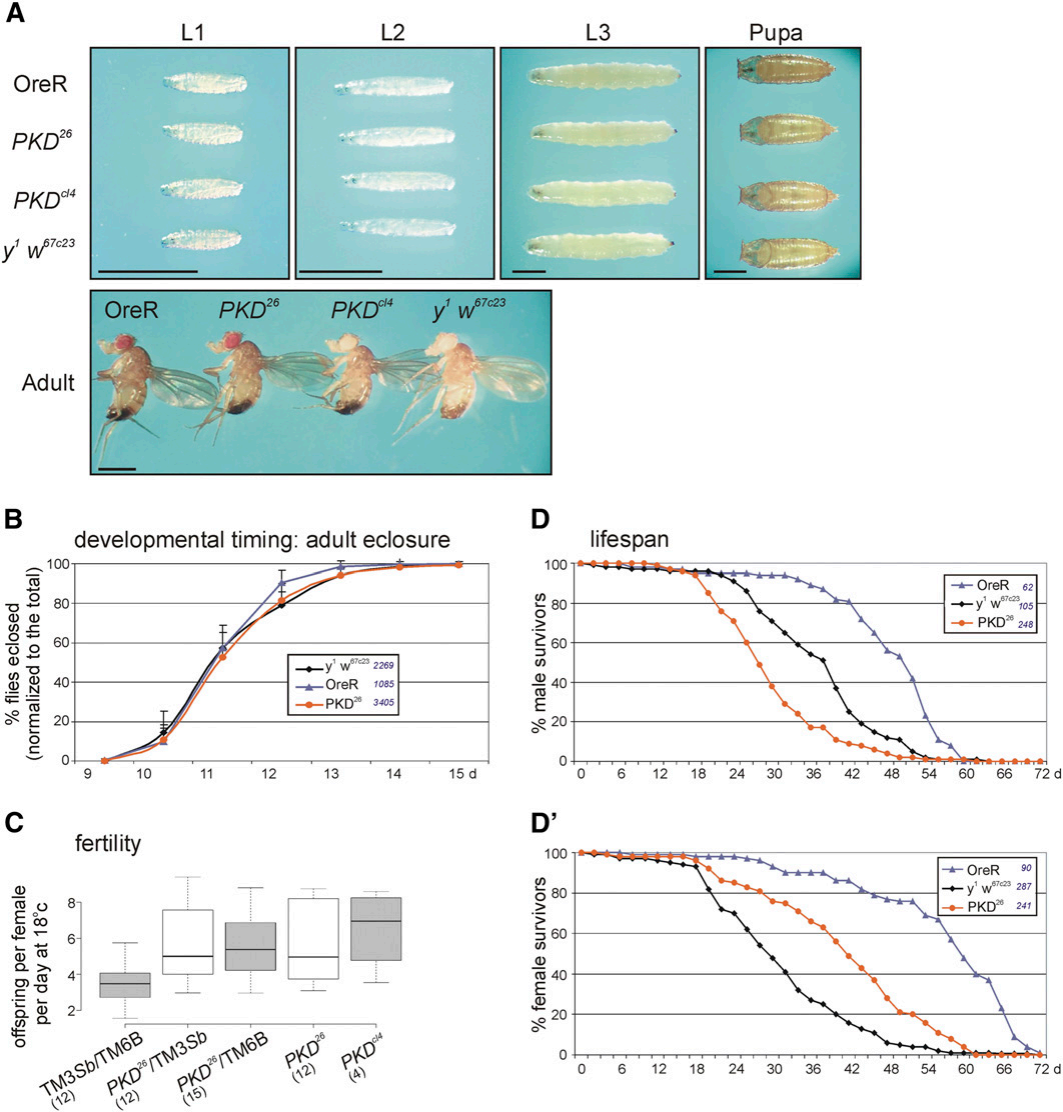
Phenotypic analyses of homozygous *PKD* mutant animals. (A) Comparison of control animals (OreR and *y^1^ w^67c23^)* with *PKD^26^* and *PKD^cl4^* mutant animal at first (L1), second (L2) and third (L3) larval stage, of pupae and of adult males. Typical examples are shown, no apparent size difference was observed. Scale bar is 1 mm in all panels. (B) Developmental timing: *PKD^26^* mutants and control flies *y^1^ w^67c23^* and OreR were reared under identical conditions at 25°C. Eclosion of adults was monitored over time (in days after egg deposition), and is shown as the fraction of the total number of animals (given in the legend). Four experiments were sampled: standard deviation is depicted as bars. Note similar timing of *PKD^26^* mutants and controls (additional controls are within supplemental Figure S2). (C) Fertility of female flies is given as offspring per female per day at 18°C (n ≥ 12 experiments as indicated; *PKD^cl4^* n = 4 experiments). Note similar fertility of homo- and heterozygous *PKD* mutants; doubly balanced flies produced least offspring. BoxPlots depict medians as Center lines; box limits indicate the 25^th^ and 75^th^ percentiles; whiskers extend 1.5 times the interquartile range. Sample points are given for each genotype in parentheses. Statistical analysis was performed with a two-tailed ANOVA test relative to TM3 *Sb* /TM6B using Dunnet’s approach (not significant). (D, D’) Lifespan of male and female control flies (OreR and *y^1^ w^67c23^) vs. PKD^26^* mutant flies is depicted. Surviving flies over time (in days after egg deposition) is shown as fraction of the total (given in the respective legend). Whereas *PKD^26^* males have a reduced life span (D), the *PKD^26^* females live slightly longer than the control *y^1^ w^67c23^* (D’). OreR flies live longest. See also supplemental Figure S3 for additional controls and statistics.

Based on our earlier studies we expected loss of PKD to affect growth and perhaps cause lethality (Maier *et al.* 2007). Yet, homozygous *PKD* null mutants were viable without apparent phenotype with respect to size or the overall pattern of the external organs (Figure 2A). Moreover, developmental timing, *i.e.*, emergence of adult flies (Figure 2B) was similar to *y^1^ w^67c23^* animals used for control, as this was the parental origin of the starting line. As the *PKD^26^* and *PKD^cl4^* mutant strains were not isogenic, however, we used additional controls to account for possible variance of the genetic background. Indeed, a great variance between the *y^1^ w^67c23^* genotype and other control strains, *i.e.*, *w^1118^*, Canton-S and notably Oregon R was observed (Figure 2, supplemental Figure S2). Still, *PKD^26^* mutants were within the normal range, and neither pupae nor adults developed particularly slower than the controls (Figure 2, supplemental Figure S2). We also addressed female fertility, which was indistinguishable between the homozygous *PKD* mutants and their heterozygous siblings (Figure 2C). As observed for fly hatching time, the life span also differed remarkably between several control strains (Figure 2D, D’; supplemental Figure S3). It was shortest in *PKD^26^* mutant males, in contrast to that of the *PKD^26^* females that lived longer than the *y^1^ w^67c23^* control (Figure 2D, D’; supplemental Figure S3).

**Figure 3:**
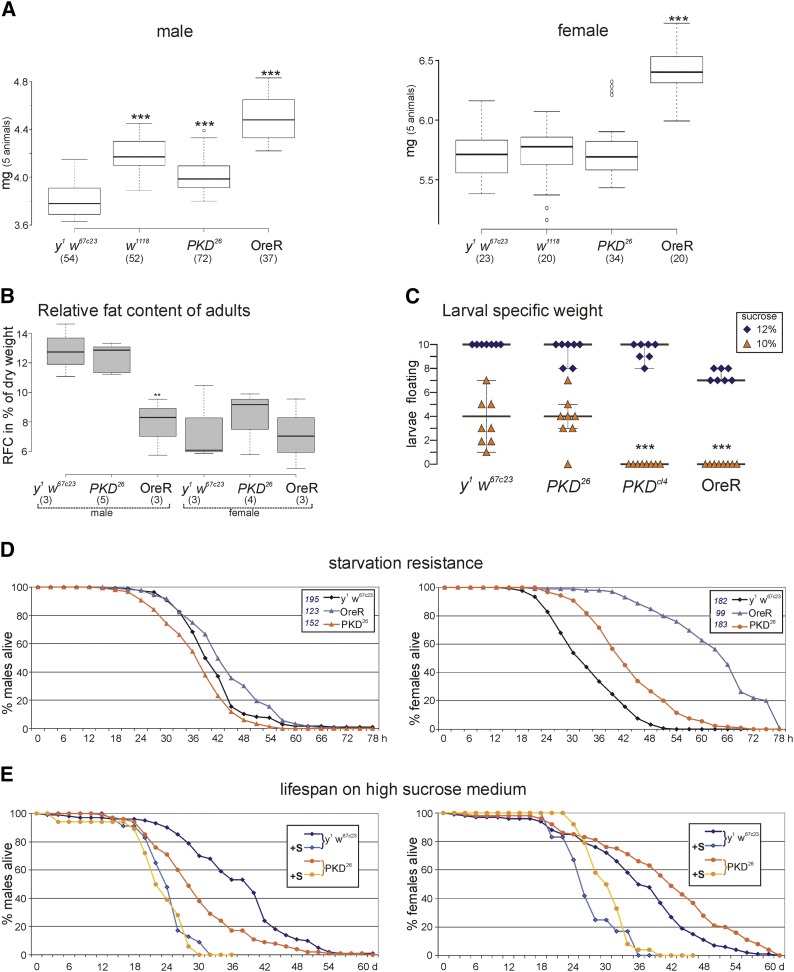
Influence of PKD on body weight and starvation resistance. (A) BoxPlot representation of the fresh weight of animals of the given genotype weighed in batches of five given in mg. The left panel shows the weight range of males, the right panel that of females. (B) The relative fat content of male and female flies of the given genotype was determined on 25-30 animals per experiment relative to the dry weight. *PKD^26^* mutants are not different from *y^1^ w^67c23^* control, whereas OreR males have a significantly larger body weight, and hence, a relatively low fat content. BoxPlots in (A) and (B) depict medians as Center lines; box limits indicate the 25^th^ and 75^th^ percentiles; whiskers extend 1.5 times the interquartile range; outliers are represented by dots. Sample points are given for each genotype in parentheses. Statistical analysis was performed with a two-tailed ANOVA test relative to *y^1^ w^67c23^* using Dunnet’s approach with ***, *P* < 0.001 and **, *P* < 0.01. (C) Larval specific weight was determined in a floating assay with sucrose of different density. Number of floating larvae is indicated for 12% sucrose (blue, 7 experiments with 10 larvae each) and 10% sucrose (orange, 8 experiments with 10 larvae each). At 8% sucrose, all larvae sank (70 animals tested per genotype). Note that *PKD^26^* resembles *y^1^ w^67c23^* control, and *PKD^cl4^* the OreR control. (D) Sensitivity to starvation stress was measured as survival of male and female flies on wet filter paper over time; dead flies were counted regularly. Whereas males of different genotype were similar, female OreR flies were highly resistant and female *y^1^ w^67c23^* very sensitive toward starvation (4-6 experiments each; the total number of tested animals is given in the legend for each genotype). (E) Lifespan was determined on medium with normal and high sucrose content (+s). High sucrose medium shortened life span of *PKD^26^* mutants as well as *y^1^ w^67c23^* control irrespective of sex to similar values.

As it was recently suggested that PKD may play a role in weight control and fat homeostasis (Ashe *et al.* 2018), we measured body weight and fat content of our mutants (Figure 3A, B). Overall, there was not a great difference between *PKD^26^* mutants and *y^1^ w^67c23^* flies, which were, however, of lower weight compared to the wild type OreR but not compared to *w^1118^* used for further control (Figure 3A, B). Moreover, OreR males, but not the females, had a significantly lower relative fat content compared to *PKD^26^* mutants and *y^1^ w^67c23^* (Figure 3B). The specific larval weight was measured in a buoyancy assay (Figure 3C): here the *PKD^26^* mutants matched the *y^1^ w^67c23^* control, whereas *PKD^cl4^* were more similar to the OreR wild type. We conclude that the *PKD* mutants lie in between the two control strains. In addition, we tested sensitivity of *PKD* mutants toward starvation under wet (Figure 3D) and dry conditions (Figure S4): males were slightly more sensitive than the *y^1^ w^67c23^* control, whereas females appeared more resistant but less than OreR (Figure 3D and supplemental Figure S4). Again, great variability toward dry starvation was observed among several strains: *y^1^ w^67c23^* and the wild type strain Canton-S were most sensitive (supplemental Figure S4). The life-shortening effect of a high-sugar diet (Magwere *et al.* 2004, Al Saud *et al.* 2015) was also observed for the *PKD^26^* mutant. When flies were raised on high sucrose, life span of *PKD^26^* mutants did not differ from *y^1^ w^67c23^* control, irrespective of sex (Figure 3E).

### PKD null mutants are sensitive for oxidative stress

**Figure 4:**
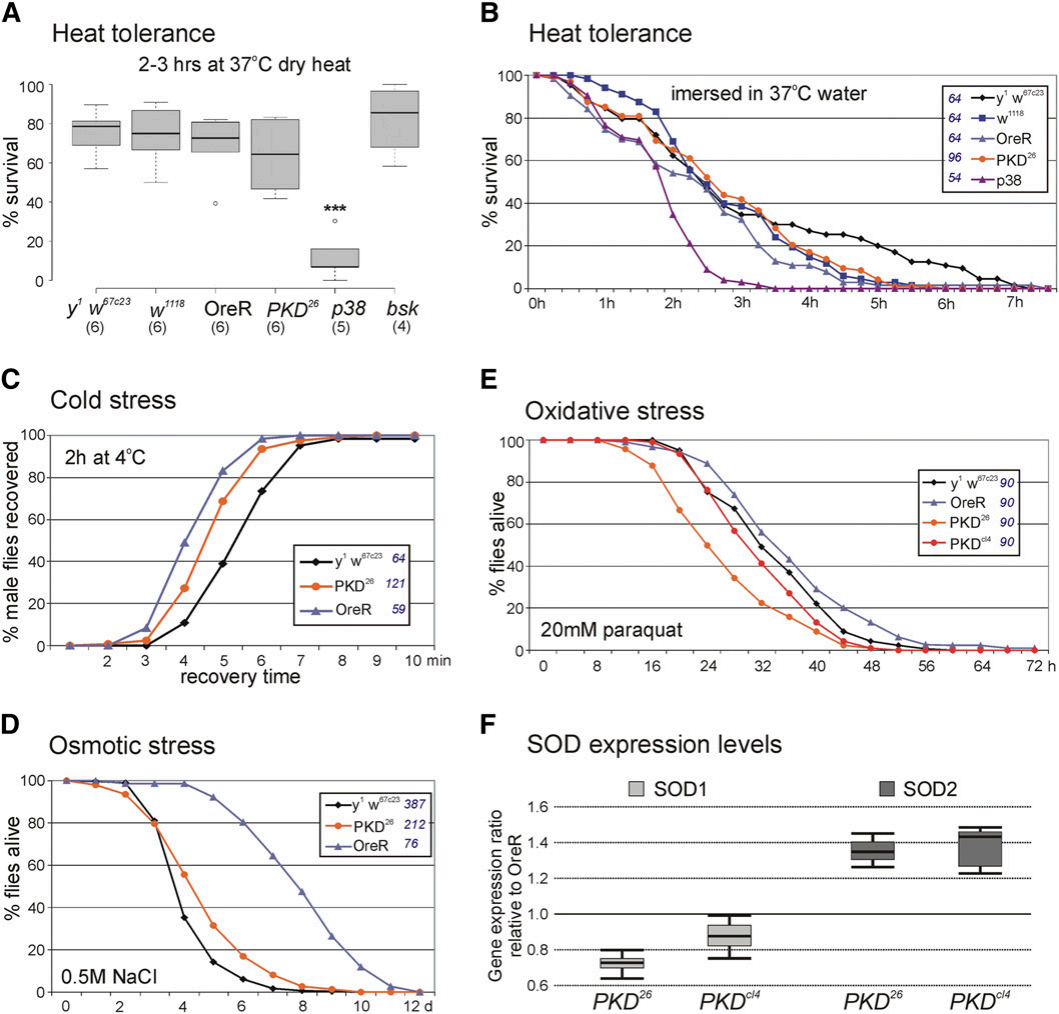
Stress resistance. Flies of the given genotype were subjected to several different stressors, exposure to dry heat (A) or wet heat (B), to cold (C), to high NaCl content for osmotic stress (D), and to oxidative stress by paraquat exposure (E). Percent of flies surviving the treatment was determined in (A) and over time in (B, D, E), respectively, whereas in (C) recovery time from treatment was measured. (A) BoxPlot representation of survivors of heat treatment is shown. Center lines show the medians; box limits indicate the 25^th^ and 75^th^ percentiles; whiskers extend 1.5 times the interquartile range; dots represent outliers. Numbers in parentheses indicate experiments with each approximately 30 flies. Statistical analysis was performed with a two-tailed ANOVA test relative to *y^1^ w^67c23^* using Dunnet’s approach with *P* < 0.001, ***. (B-E) Total number of animals tested for each genotype is given in the legend. (F) Expression levels of Cu/ZnSOD (SOD1) and of MnSOD (SOD2) were quantified by qRT-PCR in the *PKD* mutants relative to OreR control. Data were assembled from three biological and two technical replicates. Mini-max depicts 95% confidence, median corresponds to expression ratio. *Tub56D* and *Tbp* were used as reference genes. Amplification efficiencies for *SOD1* (0.92), *SOD2* (0.98), for *Tub56D* (0.97) and *Tbp* (0.96) were accounted for in determining relative quantities by REST (Pfaffl *et al.* 2002).

As PKD is apparently not strictly required for fly development, life span and fertility, we wondered whether this kinase might be involved in stress regulation. We assayed sensitivity of *PKD^26^* mutants toward a variety of stressors. *PKD^26^* mutants resisted the application of heat in a dry oven or in a water bath similar to controls (Figure 4A, B), whereas *p38a^1^* mutants were more and *bsk^1^* mutants slightly less sensitive, as described in the literature (Wang *et al.* 2003, Craig *et al.* 2004). Moreover, no particular cold sensitivity was observed (Figure 4C). Likewise, *PKD^26^* flies tolerated osmotic stress similar to the control (Figure 4D). Oxidative stress, however, was less tolerated by the *PKD* null mutant alleles compared to the *y^1^ w^67c23^* and OreR controls (Figure 4E). This is in line with the described role of mammalian PKDs to protect cells from oxidative stress mediated apoptosis. Here, PKD is involved in mitochondrial ROS detoxification by driving the expression of Manganese superoxide dismutase (MnSOD) (Storz 2007; Cobbaut and Van Lint 2018). To address a likewise role for *Drosophila* PKD, expression of superoxide dismutase was measured by quantitative RT-PCR in the two mutant *PKD* alleles. We addressed both, the expression of MnSOD (SOD2) as well as of Cu/ZnSOD (SOD1) that act in mitochondria and the cytosol, respectively, to detoxify the cell from superoxide released from mitochondria. In the absence of PKD, SOD1 and SOD2 levels were very similar those of the OreR control: a slight but not significant decrease was observed for SOD1 expression (0.72 – 0.87 fold), whereas expression of SOD2 was slightly increased (1.35-1.38 fold) (Figure 4F). Apparently, SOD regulation in *Drosophila* is largely independent of PKD activity in unstressed conditions.

### Migration of border cells and pole cells appears normal in PKD mutants

**Figure 5:**
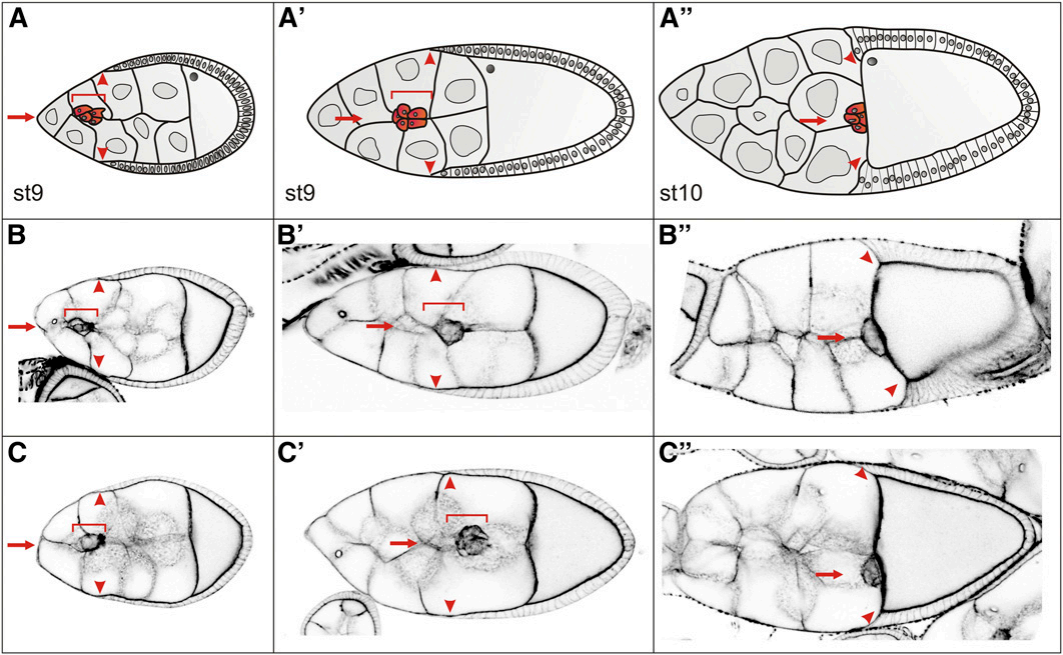
Border cell migration. (A-A’’) Sketch of border cell migration at stages 9 to 10 during *Drosophila* oogenesis. Early in stage 9, a cluster of border cells dispatches from the anterior tip of the follicle (A, red arrow), to migrate in between the large, polyploid nurse cells (A’) to finally reach the anterior of the oocyte in stage 10 (A’’). At the same time, the columnar follicle cells retract posteriorly; they are a useful marker for orderly migration (read arrowheads). Border cells are depicted in red, and highlighted by a red frame and a red arrow. (B-C’’) Cell outlines were visualized with phalloidin staining (black). Confocal images taken from the respective stages of control (B-B’’) and *PKD^cl4^* mutant females (C-C’’) are shown inverse for better visibility. Border cells are marked by red frame and arrow; columnar follicle cell margins by read arrowheads, respectively, as in (A-A’’). The migratory behavior of border cells appears not different between the two genotypes.

**Figure 6:**
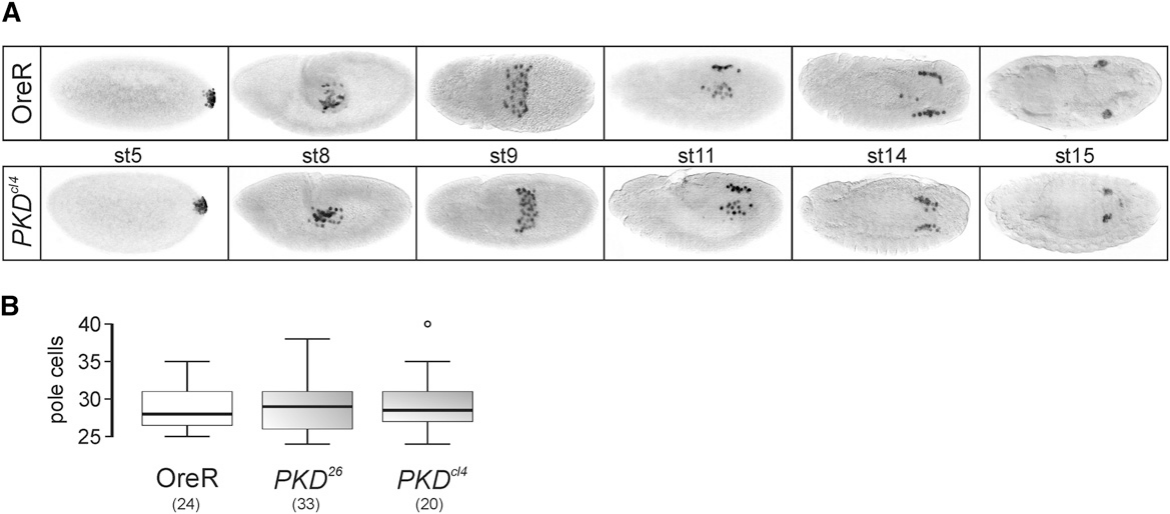
Pole cell migration. (A) Embryos were stained with anti-vasa antibodies to mark the pole cells. Anterior is to the left, embryonic stages are indicated (st5-st15). Pole cells originate at the posterior pole (stage 5), and migrate through the posterior midgut (stage 8) to move posteriorly during the following stages (9-14), before they conglomerate with mesodermal cells (stage 15) to form the round gonadal anlagen in the embryo. No apparent differences are seen between the control Oregon R (OreR, upper row) and *PKD^cl4^* mutants (lower row). (B) Moreover, the number of pole cells was not significantly different between the two *PKD* alleles, *PKD^26^* and *PKD^cl4^*, and the control OreR. Center lines of the BoxPlots show the medians; box limits indicate the 25^th^ and 75^th^ percentiles; whiskers extend 1.5 times the interquartile range; dot represents outlier. Number of data points is given in parentheses for each genotype.

Human PKDs are pivotal to cell motility by regulating cytoskeletal dynamics. Specifically, human PKDs act as negative regulator of Slingshot-phosphatase, thereby influencing cofilin availability and actin filament de/polymerization (reviewed in Olayioye *et al.* 2013). We have shown before that overexpression of active *Drosophila* PKD-SE negatively regulates Slingshot activity, suggesting a likewise involvement of PKD in cytoskeletal dynamics in the fly (Nagel *et al.* 2010, Barišić *et al.* 2011). Accordingly, we might expect an impact of a loss of *Drosophila PKD* on cell migration. We studied two well-characterized processes of cell migration during *Drosophila* development. First, we monitored migration of border cells during oogenesis. Border cells are specifically determined follicle cells that actively migrate from the anterior tip of the follicle in between the nurse cells to the anterior border of the oocyte (reviewed in Montell 2006). The timing of the migration can be followed by the columnar follicle cells that retract in parallel toward the posterior of the oocyte (reviewed in Montell 2006). No difference was seen in border cell migration behavior between *PKD^cl4^* and control (Figure 5). Second, we studied pole cell migration during embryogenesis. Pole cells are the primordial germ cells of *Drosophila*. They arise at the posterior of the embryo and migrate through the posterior midgut and along the germ band to populate the gonadal mesoderm (Hay *et al.* 1988; reviewed in Montell 2006). This process appears normal in *PKD* mutant embryos that moreover, display the same number of pole cells than the wild type (Figure 6). We conclude that PKD is not required for these two processes of cell migration in *Drosophila*. As the overexpression of an activated isoform PKD-SE can influence the cytoskeleton (Nagel *et al.* 2010, Barišić *et al.* 2011), the most likely explanation for a lack of migration defects is the presence of redundant kinases that adopt the function of PKD in its absence.

### A small candidate screen on potentially redundant kinases

The fact that a loss of *Drosophila PKD* appears to be without major phenotypic consequences suggests that it may act redundantly to some other kinase, most likely members of the family of PKC or CAMK kinases. Candidates located on the first or second chromosome were selected by the availability of mutant alleles, as shown in Supplemental Table S1, thereby covering most of the *Drosophila* PKC family members (four out of five), and about one quarter of all CAMK family members (7 of 30) (Morrison *et al.* 2000). The mutants were combined with *PKD^cl4^* to record survival rate of the offspring. The fraction of balanced *vs.* homozygous *PKD^cl4^* mutants was determined in the heterozygous background of the relevant kinase mutant. Survival rate of *PKD^cl4^* homozygotes was reduced to 70–80% in a *sqa^f01512^* and *Pkcdelta^e04408^* heterozygous background (Table 1). A likewise increased mortality of the *PKD^cl4^* homozygotes was observed when either *Pkcdelta^e04408^* or *Drak^BG00876^* were homozygous in addition (Table 1). Unfortunately, double mutants could only be tested for these two, as most of the kinase candidate mutants are homozygous lethal. As most kinase mutants are fully recessive, however, heterozygotes are unlikely to be influenced by a loss of PKD activity. Pronounced synthetic lethality, as we might have expected if one of the kinases required PKD to supplement its activity, was not observed.

**Table 1:** Candidate kinase screen Expected fraction of homozygous *PKD^cl4^* animals inferred from the number heterozygous *PKD^cl4^* siblings in a heterozygous background of the respective candidate kinase mutant is given in percent %. The final *inter se* cross was of the following genotype: M or Y/FM7; *PKD^cl4^*/TM3Sb and M/CyO; *PKD^cl4^*/ TM6B, respectively, with M representing mutant allele or deletion

Protein kinase C family			
Kinase	allele/deletion	n^a^	% PKD expected^b^
atypical protein kinase C (aPKC)	*aPKC^k06403^* / CyO	46	117.2%
Inactivation no afterpotential C (inaC)	both contained within:		
Protein C kinase 53E (PKC53E)	Df(2R)Exel6065/ CyO	75	112.5%
Protein kinase C δ(Pkcδ)	*Pkcdelta^e04408^*	84^c^	84.7%
	*Pkcdelta^e04408^* / FM7	271	85.3%
**Ca^2+^/calmodulin dependent protein kinase family (CAMK)**			
Kinase	allele/deletion	n^a^	% PKD expected^b^
AMP-activated protein kinase α subunit (AMPKα)	Df(1)Exel6227/ FM7	120	107.7%
Death associated protein kinase related (Drak)	*Drak^BG00876^*	167^c^	80.0%
	*Drak^BG00876^* / FM7	255	107.2%
loki (lok)	Df(2L)Exel7077/ CyO	221	138.6%
MAP kinase activated protein-kinase-2 (MK2)	Df(1)Exel6236/ FM7	203	94.2%
par-1 (par-1)	*par-1^k06323^* / CyO	39	139.1%
spaghetti-squash activator (sqa)	*sqa^f01512^* / CyO	151	69.6%
Stretchin-Mlck (Strn-MLCK) Myosin light chain kinase	*Strn-Mlck^c02860^* / CyO	127	134.2%

a , total number of offspring analyzed.

b , expected number of homozygous *PKD^cl4^* animals was determined from the number of the heterozygous siblings.

c , allele is homozygous viable and was tested in homozygosis.

## Discussion

We have generated specific knock out alleles of *Drosophila PKD*, which to our surprise were homozygous viable without apparent phenotype. Based on our own previous experiments, we expected conspicuous phenotypes, resulting for example from defects in the cytoskeleton or in protein secretion (Maier *et al.* 2007; Nagel *et al.* 2010; Barišić *et al.* 2011). Lack of drastic phenotypes, however, suggests redundancy: presumably other serine-threonine protein kinases of the PKC/CAMK family act in place of PKD. Our tentative candidate kinase screen indeed uncovered three kinases, Drak, Sqa and Pkcδ that may be linked with PKD activity, since respective hypomorphic alleles impeded fly viability in the absence of PKD (Table 1). The former two belong to the CAMK and the latter to the PKC family of kinases (Morrison *et al.* 2000). These results are rather intriguing in light of the known roles for mammalian PKDs in oxidative stress response and in the regulation of cell motility and invasion (Fu and Rubin 2011, Cobbaut and Van Lint 2018, Olayioye *et al.* 2013).

It is well known that mammalian PKDs oppose the apoptotic effects of oxidative stress in a variety of cells (reviewed in Storz 2007; Cobbaut and Van Lint 2018). In this process, PKD1 is activated by PKCδ in response to phospholipase D activation at the mitochondrial membrane. Consequently, active PKD1 inhibits mitochondrial depolarization and decreases the release of cytochrome C, thereby protecting cells from apoptosis, and more generally from oxidative damage. In addition, PKD1 mediates expression of MnSOD, the superoxide dismutase that detoxifies the cell from superoxides released from the mitochondria. Altogether, PKD1 activity results in pro-survival signals in oxidative stress (reviewed in Storz 2007; Cobbaut and Van Lint 2018). We have observed that *PKD^26^* mutants display an increased sensitivity toward oxidative stress (Figure 4E), whereas in unstressed conditions transcription levels of MnSOD and Cu/ZnSOD were similar to wild type (Figure 4F). In addition, downregulating the activity of the *Drosophila* PKDδ homolog strongly increased the mortality of *PKD^cl4^* homozygotes (Table 1). Together, these data strongly support a likewise protective role for *Drosophila* PKD in combating oxidative stress in the fly.

In mammals, PKDs play an important role in actin cytoskeletal dynamics via the regulation of the phosphatase Slingshot (reviewed in Olayioye *et al.* 2013). Using overexpression experiments, we have shown earlier that *Drosophila* PKD likewise affects Slingshot activity, and consequently the dynamics of filamentous actin turnover (Barišić *et al.* 2011, Nagel *et al.* 2010). However, the complete absence of PKD had no apparent influence on the migration of border or pole cells, indicating redundancy for PKD activity in the context of cell motility. A possible candidate for a redundant kinase might be Drak, which has been involved in actomyosin contractility and dynamics (Neubueser and Hipfner 2010, Chougule *et al.* 2016). Our work shows a genetic interaction between PKD and Drak, since a downregulation of Drak activity in the hypomorphic allele *Drak^BG00876^* increased mortality of *PKD^cl4^* mutants considerably (Table 1). We thus conclude that Drak and PKD may act together in the regulation of cell motility.

RNAi mediated knock down of PKD activity affected cell growth and differentiation (Maier *et al.* 2007). In accordance with these data, Ashe and co-workers reported growth defects resulting from PKD depletion (Ashe *et al.* 2018). Moreover, a specific role for PKD in the release of *Drosophila* insulin like peptide ILP2 was reported, explaining the reduced weight as well as starvation sensitivity of the *dPKD^H^* allele used in their study. The mutant *dPKD^H^* allele has a 70% reduced *PKD* mRNA level in third instar larvae (Ashe *et al.* 2018). One might have expected an even stronger phenotype in the complete absence of PKD, which however, was not observed. *PKD* null mutants were largely normal with respect to growth and weight. How can we explain the different results obtained from the null alleles *PKD^26^* and *PKD^cl4^* compared to *dPKD^H^*? First, *dPKD^H^* might rather match a null allele based on the low residual PKD expression levels. In this case, the null alleles may not show dramatically stronger phenotypes. The observed differences may then be attributed to external factors, *i.e.*, rearing conditions (food composition, relative humidity, temperature etc.) or internal factors, *i.e.*, genetic background. The latter appears more likely, as we have seen a high variance among various control strains regarding several tested parameters (Figures 2, 3, S2-S4). Still, none of the control strains displayed such strong and specific defects as the *dPKD^H^* allele in Ashe *et al.* (2018), which was developmentally retarded and underweight. We therefore favor the possibility of a second site hit in the *dPKD^H^* allele that influences its phenotype. *PKD* null mutants are evidently without or very little phenotype on their own, presumably due to the function of redundant kinase(s). Mutation in (any) one of these kinases is without conspicuous phenotype, at least when heterozygous. The absence of PKD, however, may uncover a growth defect in such a mutant, if the respective kinase is involved in the regulation for example of TOR signaling activity. A rescue of this growth defect by addition of PKD - for example by ubiquitous overexpression like Act > dPKD (Ashe *et al.* 2018) - is to be expected, since in this case, PKD can take over the redundant function. A possible candidate for such a redundant kinase might be *spaghetti squash activator* (*sqa*), which encodes a novel myosin light chain kinase with a role in starvation induced autophagy and the regulation of TOR signaling activity (Tang *et al.* 2011, Findlay *et al.* 2007). The *sqa^f01512^* allele is fully recessive, and the heterozygotes are without apparent phenotype. Still, the *sqa^f01512^* heterozygous background caused a dramatic lowered survival rate of less than 70% of *PKD^cl14^* flies (Table 1). It is hence conceivable that PKD plays indeed a role in growth regulation, however, overlaps functionally with other kinases in this process, for example with Sqa. Overall, our results suggest that presumably PKD’s absence can be replaced in the respective context by one or several other kinases with overlapping function. For example, Pkcδ has not yet been assigned a specific role, as the mutants are viable without phenotype. Still, the mutant allele *Pkcdelta^e04408^* markedly impedes fly viability in the absence of PKD activity, strongly indicating functional redundancy. For technical reasons, we could test only a small subset of the *Drosophila* CAMK family. We expect overlap of PKD with further protein kinases not yet included in this test.

To date the functions of PKDs have been studied primarily in model cell-culture systems, and only few data exist on PKDs’ functions in the context of normal cells and tissues of intact organisms (Fu and Rubin 2011). Three models have been studied by now, *D. melanogaster* (this work), *C. elegans* and the mouse. Null mutants in either of the two PKD isoforms from *C. elegans*, named DKF-1 and DKF-2, are viable. Animals lacking DKF-1 display locomotory defects, whereas DKF-2 mutants impede adult life span by affecting stress and innate immunity responses (Feng *et al.* 2006, Feng *et al.* 2007, Ren *et al.* 2009). PKD1 mutant mice are viable and fertile, but appear semilethal with only half of the expected offspring. Embryonic fibroblasts derived from these mice are highly susceptible to apoptosis induced by oxidative stress or by starvation, which is mediated by PKCδ activity (Zhang *et al.* 2015). In these cells, PKD1 is the key regulator in determining the threshold of mitochondrial depolarization affecting Bcl2-Bax fractions, implicating a role for PKD1 during aging and nutrient deprivation (Zhang *et al.* 2015). As neither PKD2 nor PKD3 can compensate the loss of PKD1, viability of the PKD1 mutants is most likely not explained by redundancy with the other PKD isoforms. Rather oxidative stress responses may be tissue specific, or defects are observed only when animals are subjected to non-laboratory, stressful conditions (Zhang *et al.* 2015). This work shows that *Drosophila PKD* null alleles are viable and fertile, however, are sensitive toward oxidative stress. Moreover, we uncovered genetic interactions of *Drosophila* PKD with three kinases PKCδ, Drak and Sqa. Most likely overlapping functions exist between PKD and other kinases of the PKC/CAMK family, explaining the lack of apparent phenotypes in the null mutants. Whether the overlap is restricted to these three kinases or extends to a larger kinase family, and whether it applies only to certain tissues or processes, will require further investigations in the future.
